# Prospective multicenter study on patient-related outcomes of orbital floor reconstruction using ultrahigh-molecular-weight polyethylene

**DOI:** 10.1371/journal.pone.0359326

**Published:** 2026-10-08

**Authors:** Stefanie Wilhelm, Raimund Preidl, Konstantinos Afxentiou, Marco Kesting, Adem Aksu, Rainer Lutz, Lucas Ritschl, Katharina Pippich, Achim von Bomhard

**Affiliations:** 1 Technical University of Munich, Munich, Germany; 2 Department of Oral and Maxillofacial Surgery, University of Erlangen-Nuremberg, Erlangen, Germany; 3 Quantum, Private Practice, Heilbronn, Germany; 4 KLS Martin SE & Co. KG, Tuttlingen, Germany; 5 Department of Oral and Cranio-Maxillofacial Surgery, Faculty of Medicine, UKGM GmbH, University Hospital Marburg, Marburg, Germany; 6 Philipps-Universität Marburg, Marburg, Germany; 7 Department of Maxillofacial Surgery, Technical University of Munich, Munich, Germany; 8 INN TAL MKG, Private Practice, Rosenheim, Germany; Universidade de Trás-os-Montes e Alto Douro: Universidade de Tras-os-Montes e Alto Douro, PORTUGAL

## Abstract

This prospective, multicenter clinical study evaluated patient-reported outcomes, functional recovery, and quality of life following orbital floor reconstruction using ultra-high-molecular-weight-polyethylene (UHMWPE) implants. The study emphasized both subjective recovery and the clinical performance of the implant material. Between June 2022 and July 2023, 46 adult patients (≥ 18 years of age) with radiologically confirmed orbital floor fractures requiring surgical reconstruction were enrolled and completed the full follow-up protocol. Surgical reconstruction was performed according to standardized procedures using UHMWPE implants. Pre- and postoperative CT or CBCT imaging was obtained when clinically indicated; radiological outcomes of this cohort have been reported separately. Pre- and postoperative evaluations included an assessment of pain, swelling, ocular motility, diplopia, sensory function, foreign body sensation, overall satisfaction and orbital volume, which was measured using three-dimensional imaging. Intraoperative parameters were also recorded. Patient-reported outcomes were collected through validated questionnaires and visual analogue scales, repeated at each visit, follow-up examinations were performed approximately one day, ten weeks, six months, and twelve months after surgery. Overall, clinical and patient-reported outcomes improved over the follow-up period. Pain intensity declined markedly within the first week after surgery and remained consistently low thereafter. Swelling, numbness, and the sensation of a foreign body gradually subsided, with almost complete resolution after one year. Furthermore, imaging confirmed stable anatomical reconstruction without implant displacement or volume loss. In general patient satisfaction increased steadily throughout the observation period, reaching near-maximal levels after twelve months. No cases of infection, inflammatory reaction or implant-related complication were recorded. In this prospective cohort, orbital floor reconstruction using UHMWPE was associated with favorable functional, aesthetic and patient-reported recovery over 12 months. These findings support the value of incorporating patient-centered outcomes into the assessment of recovery after orbital trauma, while comparative effectiveness and long-term safety require further controlled studies.

## Introduction

The orbital region is frequently affected by facial trauma due to its delicate bony and soft-tissue anatomy [1]. Orbital fractures are common and clinically significant injuries that require a timely diagnosis. When necessary, they require precise surgical repair to restore orbital volume and soft-tissue support, and to reduce the risk of persistent diplopia and enophthalmos [2,3]. Orbital fractures are prevalent among patients with facial trauma ranges from 10–25%, which emphasizes their significance in maxillofacial surgery [4].

In recent decades, various materials have been used for orbital floor reconstruction, including autologous bone grafts, titanium meshes and resorbable polymers. Although autologous grafts are biocompatible, they are limited by donor-site morbidity and availability [5]. Consistent goals across techniques include restoring anatomy and volume, supporting soft tissue and minimizing complications, while balancing biocompatibility, availability and intraoperative adaptability [5,6].

Pragmatic classifications distinguish between resorbable and non-resorbable, porous and non-porous, standard and patient-specific and autologous/allogenic/xenogenic and alloplastic solutions [7,8].

Resorbable polymers are appealing because they do not leave behind any permanent material and can be easily molded. Clinical studies support their use, particularly for small defects. However, mechanical support declines as the material resorbs [8–10].

Non-resorbable materials, such as titanium meshes, provide long-lasting stability for larger defects. While they provide robust structural support, they may be associated with soft-tissue adherence and motility restriction [11,12]. Furthermore, they are more challenging to adapt to the defect during surgery.

Porous implants enable fibrovascular ingrowth, which can improve secondary stability and prevent migration [13,14]. However, delayed inflammatory events are possible and require monitoring [8]. Although, patient-specific implants can improve anatomical precision compared to standard preformed plates, however higher manufacturing complexity and costs must be considered [8,15].

Although autologous grafts are the gold standard in terms of biocompatibility, they are limited by donor-site morbidity and availability. This has led to alloplasts being adopted more widely in routine care [5,6,8].

Recent advances in materials science have encouraged the exploration of innovative biomaterials for orbital reconstruction. Ultra-high-molecular-weight polyethylene (UHMWPE) has emerged as a promising alternative to conventional materials, due to its high wear resistance, mechanical strength and biocompatibility. It’s very long polymer chains (approximately 3.5–6 million g/mol) contribute to its low coefficient of friction. In orbital reconstruction, it enables fibrovascular ingrowth, improving secondary stability while maintaining sufficient rigidity to support the contents of the orbit [14,16,17].

Overall, the choice of implant material for orbital reconstruction remains highly indication-independent, with long-term functional and subjective outcomes being a particular area of debate [5,7,8].

Although previous studies have demonstrated favorable clinical and radiological outcomes following orbital floor reconstruction, patient-reported outcomes remain comparatively underreported. Most available studies primarily focus on anatomical restoration, ocular motility, implant-related complications, and radiological findings, whereas patient’s perceptions of functional recovery, facial appearance, pain, and quality of life have received considerably less attention. As clinical success may not fully reflect patients perceived recovery, the present prospective, multicenter clinical study was designed to assess patient-reported outcomes, functional recovery, and overall quality of life following orbital floor reconstruction using UHMWPE implants. By integrating standardized clinical assessments with validated patient questionnaires, the study provides a comprehensive evaluation of both physical and psychosocial recovery. This approach offers insight into the safety and effectiveness of UHMWPE as a reconstructive material in orbital trauma surgery, as well as its patient-centered benefits. The radiological and anatomical outcomes of this patient cohort, including orbital volume restoration, have been reported previously [18].

## Materials and methods

### Ethical approval and study design

This prospective multicenter study was conducted between June 2022 and July 2023. Ethical approval was obtained from the Ethics Committee of the Friedrich-Alexander-University Erlangen-Nürnberg (approval no.: 21–423-B), the Technical University of Munich (approval no.: 737/21 S) and the State Medical Chamber of Baden-Württemberg (approval no. B-F-2022–031).

Written informed consent was obtained from all participants before inclusion. The study was designed to evaluate clinical, functional, and patient-reported outcomes following reconstruction of the orbital floor with ultra-high-molecular-weight polyethylene (UHMWPE) implants.

### Patient selection

From June 2022 to July 2023, adult patients with a radiologically confirmed orbital floor fracture requiring surgical reconstruction were assessed for eligibility at the participating centers. Inclusion required capacity to provide informed consent, a physician-confirmed indication for orbital floor reconstruction using the UHMWPE study implant and written informed consent.

Patients were excluded if they had any of the following: ophthalmological or neurological conditions affecting ocular motility (1), severe systemic diseases (2), prior orbital surgery or radiotherapy in the orbital region (3), current immunosuppressive therapy (4), or a history of substance or alcohol abuse (5). Pregnant women (6) or individuals planning to become pregnant during the study period (7), as well as participants involved in other clinical or medical device trials (8) were also excluded.

A total of 46 patients who fulfilled all the inclusion criteria and were enrolled consecutively. All of them underwent a standardized preoperative assessment including a medical history, ophthalmological and clinical examinations, and three-dimensional imaging.

The final sample size was determined by considering the anticipated dropout rate and the feasibility of follow-up at each site. The patient selection is summarized in Fig 1.

**Fig 1:**
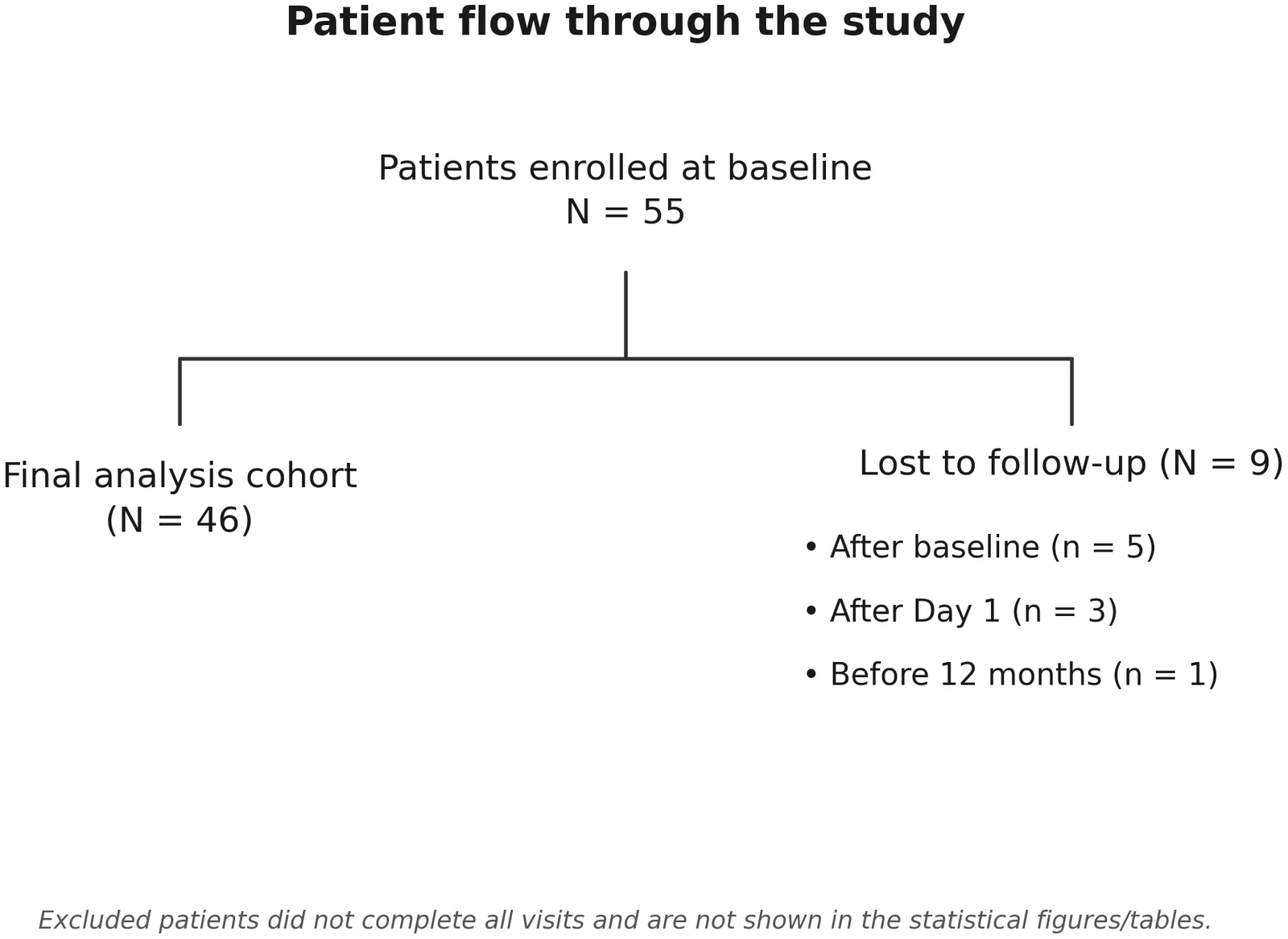
Flow diagram of patient enrollment and inclusion in the final analysis.

### Surgical procedure

All surgical interventions were performed under general anesthesia by craniofacial surgeons who were certified by the relevant board and experienced in managing craniofacial trauma. Standardized operative protocols were applied at all participating centers. The choice of surgical approach (transconjunctival or transcutaneous) depended on fracture configuration, degree of swelling and individual anatomical factors.

Following exposure of the orbital floor, the herniated soft tissue was repositioned, and the fracture margins were defined. UHMWPE implants were trimmed intraoperatively to ensure precise anatomical adaptation. The UHMWPE implant was individually trimmed to match the size and shape of the orbital defect. Implant stabilization was achieved without additional fixation by press-fit placement. Correct implant positioning and defect coverage were verified intraoperatively by direct visual inspection.

The following intraoperative data were documented: approach type, duration, defect size, implant dimensions, bleeding and complications. Hemostasis was achieved in all cases. Patients received standardized antibiotics and analgesia in accordance with institutional protocols. They were closely monitored for complications postoperatively and underwent routine follow-up.

### Postoperative management and follow-up

Clinical and patient-reported evaluations were conducted at four defined postoperative intervals: one day, ten weeks ± four weeks, six months ± two months, and twelve months ± two months after surgery.

At each visit, patients completed an identical questionnaire designed to enable direct comparison between the different time points. The questionnaire covered general health status, symptoms related to the surgery, subjective aesthetic perception, emotional and physical limitations and overall satisfaction.

In addition, the investigator performed a standardized clinical examination at every follow-up, assessing external appearance, ocular motility, and the infraorbital nerve sensitivity.

The analysis included both objective parameters and patient-reported outcomes (PROs) to provide a comprehensive overview of postoperative recovery and functional, aesthetic, and psychosocial aspects.

Preoperative and postoperative three-dimensional imaging was performed using computed tomography (CT). Three-dimensional segmentation analysis was used to assess orbital volume restoration and anatomical reconstruction. Routine postoperative CT examinations were performed as part of the study protocol. The condition of the maxillary sinus was not assessed according to a dedicated standardized sinus protocol. Additional assessment was performed only in patient with corresponding clinical symptoms. Because the maxillary sinus was assessed only when clinically indicated, asymptomatic sinus abnormalities may have remained undetected.

All findings and patient responses were recorded using the Climedo Health App.

### Data collection and statistical analysis

Data were collected and managed using the electronic case report system Climedo Health App (Climedo Health GmbH, Germany) and Microsoft Excel (Microsoft Corporation, Redmond, WA, USA). Descriptive statistics of the collected clinical and patient-reported data were performed in R (R Foundation for Statistical Computing, Vienna, Austria, Version 4.4.2, 2024-10-31) using the tidyverse, readxl, nlme, ggplot2 and lme4 packages.

Continuous and ordinal patient-reported outcomes were summarized at each time point as mean ± standard deviation (SD) and median with interquartile range (IQR), together with the number of available observations. Because the same patients were assessed repeatedly at five time points (pre-operative, day 1, 10 weeks, 6 months and 12 months), changes over time were analyzed for each endpoint using a linear mixed-effects model with time point as a fixed factor and a random intercept per patient, thereby accounting for the within-patient correlation of repeated measurements and using all available observations under a missing-at-random assumption. The overall effect of time was tested with a Type III F-test; estimated marginal means with 95% confidence intervals and contrasts of each post-operative time point versus baseline were derived from the model, and standardized effect sizes (paired Cohen’s dz) were computed for each post-operative time point versus baseline.

Normality of the model residuals was assessed using the Shapiro–Wilk test and quantile–quantile plots. Because several outcomes were bounded ordinal scales with pronounced floor or ceiling effects, results were confirmed in sensitivity analyses using the non-parametric Friedman test with Wilcoxon signed-rank post-hoc comparisons; for the ordinal general-health item a cumulative link mixed model was additionally fitted. All three approaches yielded concordant results.

Missing questionnaire items (present only at the 10-week visit, 5/46 patients) were assumed to be missing at random and were handled within the mixed model using all available observations; a complete-case analysis (n = 41) gave concordant results.

All tests were two-sided with a significance level of 0.05. As the patient-reported outcomes were pre-specified secondary endpoints, analyses were exploratory: primary contrasts are reported unadjusted and, for transparency, Holm-adjusted p-values are additionally provided; exact p-values are reported throughout.

No formal a priori power calculation was performed for the patient-reported outcomes. The sample size was determined by feasibility and expected recruitment capacity at the participating centers. As the patient-reported outcomes were pre-specified secondary endpoints, the corresponding analyses were considered exploratory.

### Material

Ultra-high-molecular-weight polyethylene (UHMWPE) was used for orbital reconstruction. Its superior mechanical properties are due to its long polymer chains and high degree of polymerization. Compared to conventional polyethylene, UHMWPE demonstrates greater strength, wear resistance and dimensional stability [19,20].

Furthermore, it offers enhanced mechanical stability while permitting more precise volumetric adaptation compared to titanium mesh [21]. However, oxidative degradation may be promoted by oxygen exposure and radiation sterilization and may lead to the formation of wear particles with osteolytic potential [22]. To enhance material stability, a series of modified irradiation and surface treatment techniques have been developed [20]. During the study period, other reconstructive materials were also available at the participating centers. However, patients enrolled in the present study underwent UHMWPE reconstruction because use of the study implant was predefined by the protocol.

## Results

Of the 46 patients who met the inclusion criteria, 25 were male (54.3%) and 21 were female (45.7%). The mean age of the male patients was 49.5 years (SD = 20.1), ranging from 21 to 97 years. The age range of the female patients was 52–88 years, and they were, on average, older, with a mean age of 68.5 years (SD = 11.1). When analyzed irrespective of sex, the mean age of the overall study population was 58.2 years (SD = 19.0).

Most fractures (58.7%) occurred on the right side, 37% on the left, and 4.3% were bilateral. Falls were the most common cause of injury (52.2%), followed by traffic accidents (21.7%) and sports-related incidents (10.9%). Other mechanisms included miscellaneous accidents (8.7%) and interpersonal violence (6.5%).

Preoperative assessment revealed hematomas of varying intensity in 41 patients (89.1%), and 27 patients (58.7%) reported hypesthesia in the infraorbital nerve distribution area. Three patients had restricted ocular mobility, two experienced diplopia and none had enophthalmos.

Postoperative recovery was favorable across all parameters. On the first day after surgery, 42 patients (91.3%) exhibited local swelling and/or irritation. These symptoms progressively subsided, with 39 of 41 patients with available clinical findings (95.1%) showing normal local findings after ten weeks. Local clinical findings had normalized in all patients at the twelve-month follow-up.

Ten patients (21.7%) reported a foreign-body sensation on the first postoperative day. This symptom showed mild persistence after ten weeks, with the condition still present in two patients (4.3%) at the twelve-month follow-up, but with only minimal intensity.

Hypesthesia was present in 25 patients (54.3%) on the first day after surgery. This symptom gradually improved over time, persisting in four patients (8.7%) after one year.

Diplopia, which was observed preoperatively in two patients, persisted postoperatively after one year in one case (2.2%).

Twenty-five patients (54.3%) reported postoperative pain and discomfort on the first day after surgery, three patients (6.5%) reported it after six months, and none reported it after one year.

Pain intensity (visual analogue scale, 0–10) changed significantly over the follow-up period (linear mixed-effects model and Friedman test, both p < 0.001). Compared with baseline, there was a small transient increase on the first postoperative day (+0.48; p = 0.016; dz = 0.31), after which pain decreased markedly to 0.2 at ten weeks and to 0.0 at twelve months. Each time point from ten weeks onward was substantially lower than baseline (−1.96 to −2.15 points; all p < 0.001; large effect sizes, dz = −1.32 to −1.53) (Fig 2).

**Fig 2:**
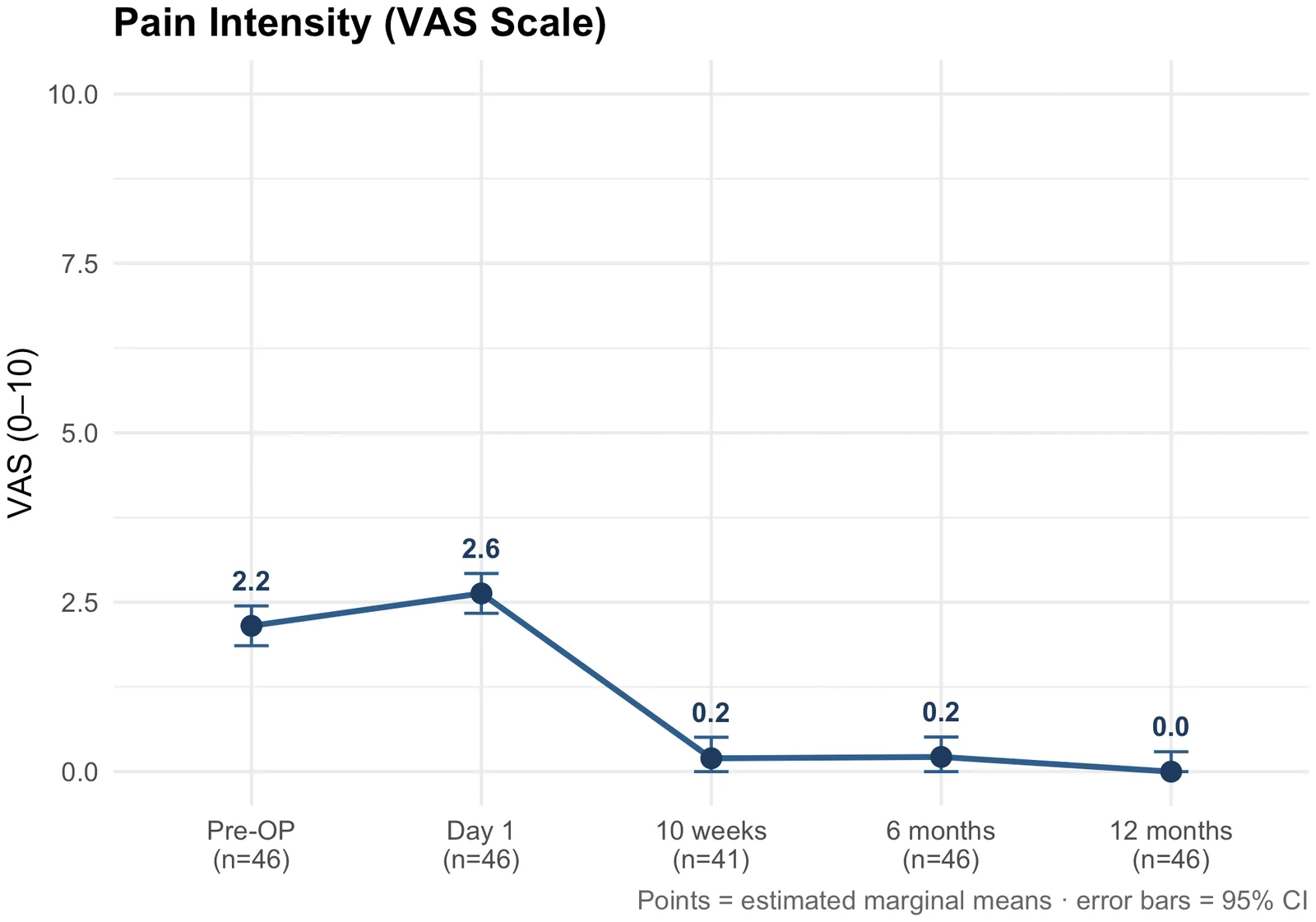
Pain intensity (VAS Scale) over the follow-up period (VAS 0–10). Points represent estimated marginal means derived from the linear mixed-effects model; error bars indicate 95% confidence intervals.

Self-perception (ANA scale) improved significantly over time (both p < 0.001), rising from a mean of 5.8 pre-operatively to 8.0 at twelve months; the improvement was significant from ten weeks onward (+1.06, p=0.003 at 10 weeks; +2.22, p<0.001 at 12 months) (Fig 3).

**Fig 3:**
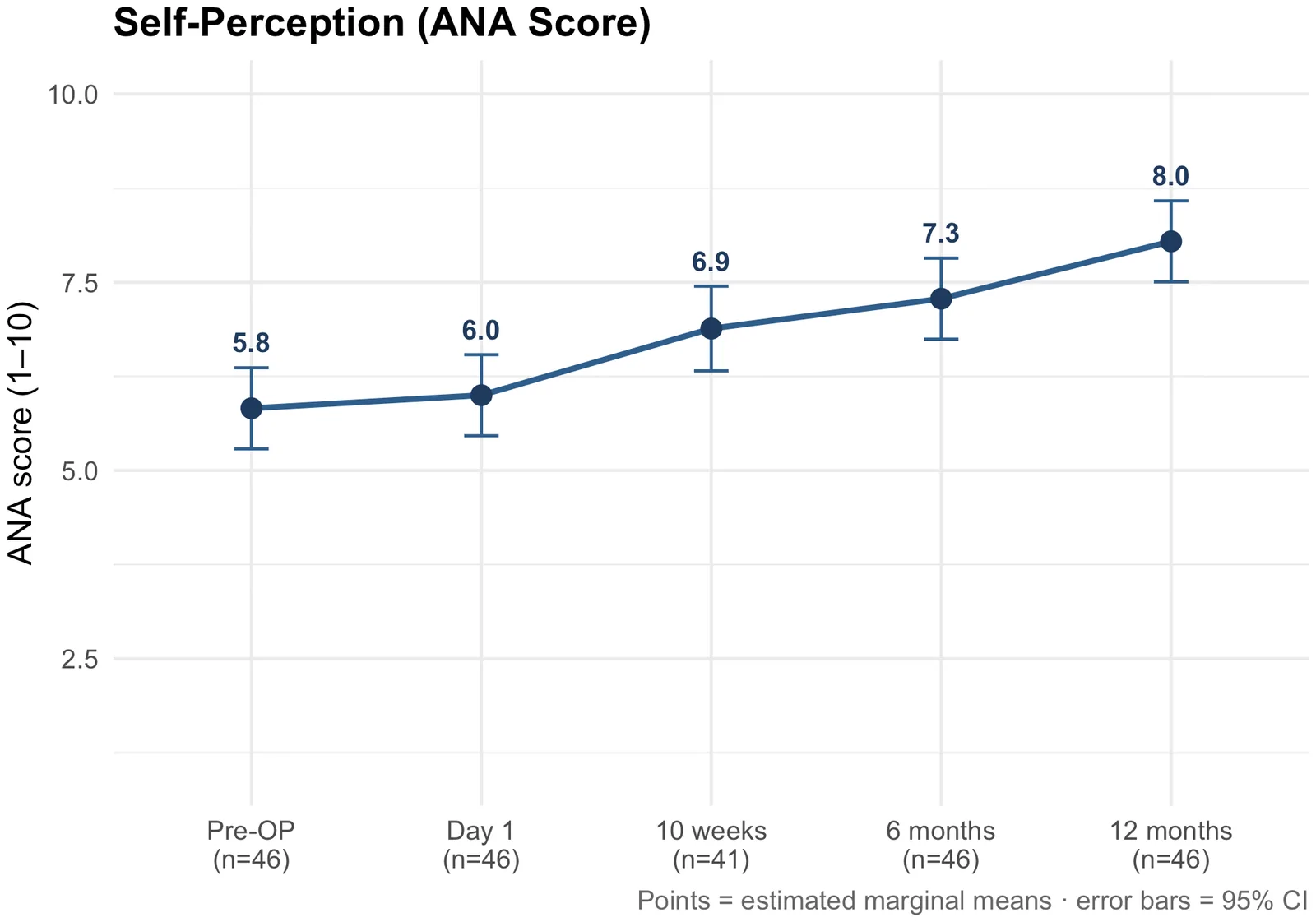
Self-perception over the follow-up period (ANA scale). Points represent estimated marginal means derived from the linear mixed-effects model; error bars indicate 95% confidence intervals.

Treatment satisfaction increased significantly (both p < 0.001), from a mean of 7.3 pre- operatively to 9.6 at twelve months, with significant gains from the first postoperative day onward (all p ≤ 0.001) (Fig 4).

**Fig 4:**
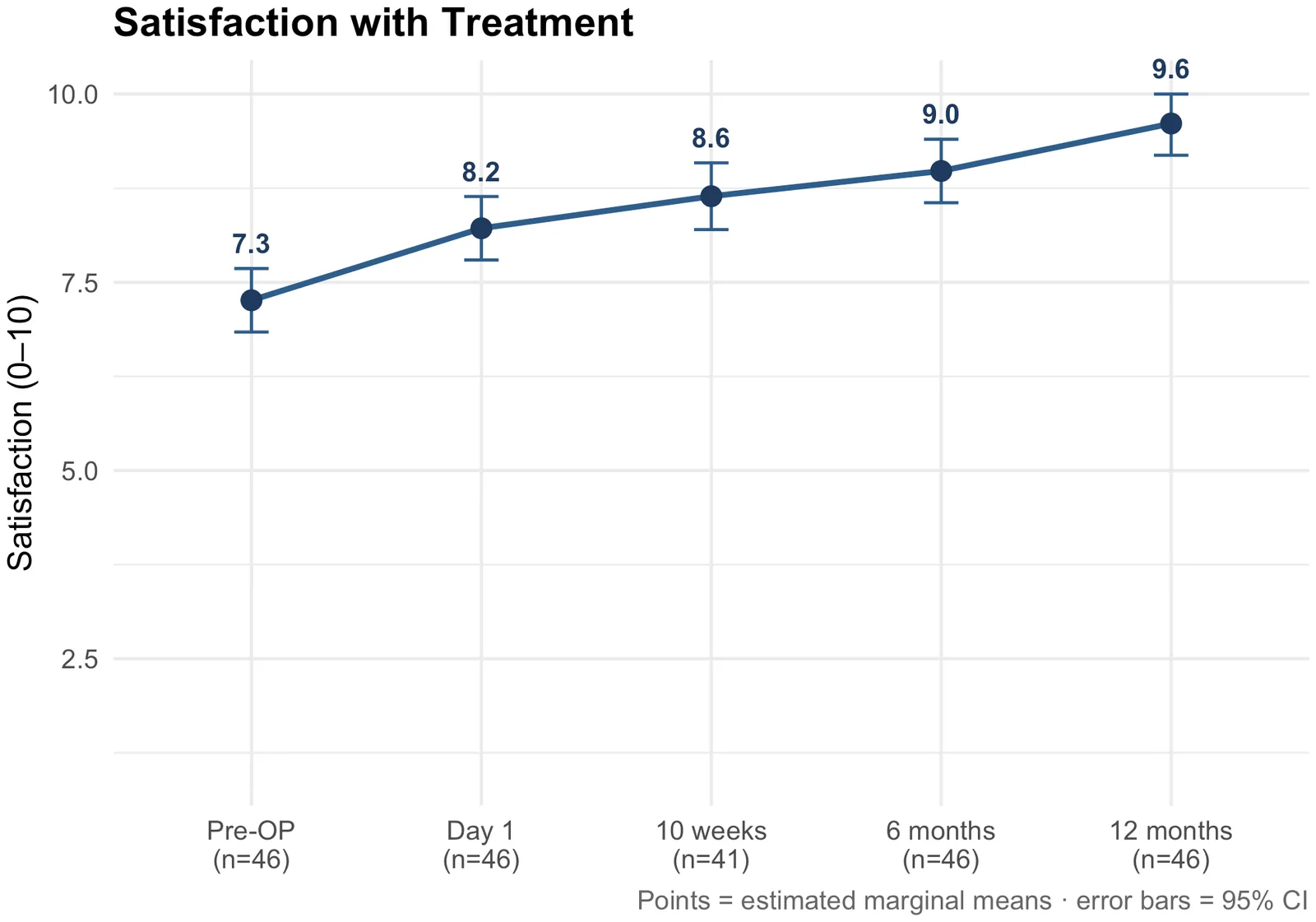
Satisfaction with treatment over the follow-up period (0–10 scale). Points represent estimated marginal means derived from the linear mixed-effects model; error bars indicate 95% confidence intervals.

These findings were supported by improvements in social and emotional dimensions. Initially, some patients reported reductions in social interaction due to physical or emotional strain, and these limitations decreased substantially during follow-up.

## Discussion

Our study population was almost equally gender-balanced, with a slight predominance of male patients. The mean age was 58.2 years, which is higher than in comparable studies [23,24]. In contrast to previous reports, the right orbit was more frequently affected in our study [25,26]. Falls were the main cause of injury, whereas violent impacts were more commonly reported in other studies. This discrepancy may be related to the higher average age of our cohort, since older patients are more prone to falls [27], whereas younger individuals are more likely to be involved in physical altercations.

As only adults were included, the findings cannot be extrapolated to pediatric or skeletally immature patients. Orbital reconstruction in children requires particular consideration of ongoing craniofacial growth, fracture pattern and the long-term behavior of permanent alloplastic implants. Previous evidence indicates that treatment outcomes may differ between adult and pediatric patients, supporting the separate evaluation of these population [28]. The present study provides no evidence regarding the safety or suitability of UHMWPE in growing patients. Therefore, its use in pediatric orbital reconstruction should be evaluated separately.

The most frequent preoperative symptoms in our cohort were hematoma and diplopia; previous reports have also described these as common findings [29,30]. Preoperative enophthalmos was not observed in our patients, despite it being described as a common accompanying symptom in other studies [31,32]. This absence may be explained by perioperative swelling, which could have masked or temporarily reduced the appearance of enophthalmos during the preoperative assessment.

Following surgery, patients primarily exhibited symptoms such as swelling, hematoma, sensory disturbances and diplopia. These findings largely align with the complication patterns described in the literature, emphasizing the importance of meticulous intraoperative assessment to prevent functional impairment [33,34].

In line with previous findings, our study also showed that sensory disturbances improve over time [35]. The initially high rate of hypoesthesia was probably due to intraoperative manipulation and localized swelling. Over time, there was a significant improvement in symptoms. This pattern is consistent with gradual recovery of intraorbital sensory function. Furthermore, the literature indicates that early surgical intervention is associated with a lower rate of postoperative complications, which is consistent with our observations.

The present findings demonstrate an improvement in health-related quality of life following orbital surgery. Functional and subjective recovery was observed within the first few months after surgery, approaching near-normalization by six months. These observations are consistent with previous reports and emphasize the effectiveness of surgical reconstruction [36,37].

Psychosocial and emotional improvements occurred alongside physical recovery. As physical healing progressed, social withdrawal and emotional distress declined concomitantly, accompanied by an improvement in self-perception. These developments were accompanied by a steady reduction in pain intensity, which decreased rapidly after an initial postoperative peak. These findings suggest that effective pain management and an uncomplicated recovery were achieved.

The positive trend was further substantiated by the aesthetic outcomes, demonstrating improvements in facial appearance and self-image, resulting in elevated overall satisfaction.

These favorable clinical outcomes should be interpreted in the context of currently available orbital reconstruction materials. Titanium mesh provides excellent mechanical stability, particularly in larger defects but may be associated with more challenging intraoperative adaption and soft-tissue adherence [8,11]. Resorbable polymers are mainly suitable for smaller defects because of their gradual loss of mechanical strength [6,8]. Porous polyethylene implants are characterized by fibrovascular tissue integration and good biocompatibility [13,14]. In the present cohort, no inflammatory reactions or implant-related complications were recorded during the 12-month follow-up; radiological and anatomical outcomes have been reported separately [18]. However, long-term complications and cost-effectiveness were not assessed and as no alternative implant materials were evaluated, direct comparisons cannot be made. Future comparative studies with longer follow-up are warranted.

Despite these encouraging findings, several limitations of the present study should be acknowledged. The sample size was relatively small and the follow-up period was limited to 12 months, precluding conclusions regarding rare or long-term complications. Furthermore, all patients underwent reconstruction with UHMWPE implants according to the predefined study protocol, therefore no direct comparison with alternative reconstruction materials was possible.

In summary, the findings suggest that the restoration of quality of life following orbital fracture repair is the result of a complex interplay of functional, aesthetic, and psychosocial factors. Successful orbital reconstruction restores anatomical integrity and functional capacity and promotes lasting improvements in overall well-being and social reintegration.

## Conclusion

The present study evaluated the use of ultra-high-molecular-weight polyethylene (UHMWPE) for orbital floor reconstruction. In this prospective cohort, orbital floor reconstruction using UHMWPE was associated with favorable functional, aesthetic and patient-reported outcomes over a 12-month follow-up period. No implant-related complications were observed in the present sample. However, the absence of a comparator group, the modest sample size and the limited follow-up preclude definitive conclusions regarding comparative safety or effectiveness. Larger controlled studies with a longer follow-up period are required.

## Supporting information

S1 File Minimal anonymized dataset. (XLSX)

S2 File Figure data. (XLSX)

S3 File Results tables. (XLSX)

## References

[1] Reith W, Yilmaz U. Orbita. In: Delorme S, Reimer P, Reith W, Schäfer-Prokop C, Schüller-Weidekamm C, Uhl M. Weiterbildung Radiologie: CME-Beiträge aus: Der Radiologe Januar 2015 – Dezember 2015. Berlin, Heidelberg: Springer Berlin Heidelberg; 2017. p. 89–106.

[2] Boyette JR, Pemberton JD, Bonilla-Velez J. Management of orbital fractures: Challenges and solutions. Clin Ophthalmol. 2015;9:2127–37. doi: 10.2147/OPTH.S80463 26604678 PMC4655944

[3] Cole P, Boyd V, Banerji S, Hollier LHJ. Comprehensive management of orbital fractures. Plastic and Reconstructive Surgery. 2007;120(7):57S-63S. 10.1097/01.prs.0000260752.20481.b4 18090729

[4] Roth FS, Koshy JC, Goldberg JS, Soparkar CNS. Pearls of orbital trauma management. Semin Plast Surg. 2010;24(4):398–410. doi: 10.1055/s-0030-1269769 22550464 PMC3324224

[5] Mok D, Lessard L, Cordoba C, Harris PG, Nikolis A. A review of materials currently used in orbital floor reconstruction. Can J Plast Surg. 2004;12(3):134–40. doi: 10.1177/229255030401200302 24115885 PMC3792801

[6] Avashia YJ, Sastry A, Fan KL, Mir HS, Thaller SR. Materials used for reconstruction after orbital floor fracture. J Craniofac Surg. 2012;23(7 Suppl 1):1991–7. doi: 10.1097/SCS.0b013e31825aada1 23154365

[7] Totir M, Ciuluvica R, Dinu I, Careba I, Gradinaru S. Biomaterials for orbital fractures repair. J Med Life. 2015;8(1):41–3. 25914737 PMC4397518

[8] Dubois L, Steenen SA, Gooris PJJ, Bos RRM, Becking AG. Controversies in orbital reconstruction-III. Biomaterials for orbital reconstruction: A review with clinical recommendations. Int J Oral Maxillofac Surg. 2016;45(1):41–50. doi: 10.1016/j.ijom.2015.06.024 26250602

[9] Gierloff M, Seeck NGK, Springer I, Becker S, Kandzia C, Wiltfang J. Orbital floor reconstruction with resorbable polydioxanone implants. J Craniofac Surg. 2012;23(1):161–4. doi: 10.1097/SCS.0b013e3182413edc 22337397

[10] Dacho A, Steffen H, Ziegler CM, Conradt C, Dietz A. Orbital floor reconstruction. Comparison of a new perforated 0.15 mm thick PDS layer with established titanium dynamic mesh. HNO. 2002;50(1):21–8. doi: 10.1007/s106-002-8061-8 11963783

[11] Ellis E, Messo E. Use of nonresorbable alloplastic implants for internal orbital reconstruction. J Oral Maxillofac Surg. 2004;62(7):873–81. doi: 10.1016/j.joms.2003.12.025 15218569

[12] Lee HH, Alcaraz N, Reino A, Lawson W. Reconstruction of orbital floor fractures with maxillary bone. Arch Otolaryngol Head Neck Surg. 1998;124(1):56–9. doi: 10.1001/archotol.124.1.56 9440781

[13] Berghaus A. Porous polyethylene in reconstructive head and neck surgery. Arch Otolaryngol. 1985;111(3):154–60. doi: 10.1001/archotol.1985.00800050048005 3977737

[14] Lin I-C, Liao S-L, Lin LLK. Porous polyethylene implants in orbital floor reconstruction. J Formos Med Assoc. 2007;106(1):51–7. doi: 10.1016/S0929-6646(09)60216-3 17282971

[15] Zimmerer RM, Ellis E, Aniceto GS, Schramm A, Wagner MEH, Grant MP, et al. A prospective multicenter study to compare the precision of posttraumatic internal orbital reconstruction with standard preformed and individualized orbital implants. J Craniomaxillofac Surg. 2016;44(9):1485–97. doi: 10.1016/j.jcms.2016.07.014 27519662

[16] Bracco P, Bellare A, Bistolfi A, Affatato S. Ultra-high molecular weight polyethylene: Influence of the chemical, physical and mechanical properties on the wear behavior. A review. Mater. 2017;10(7). 10.3390/ma10070791 PMC5551834 28773153

[17] Hussain M, Naqvi RA, Abbas N, Khan SM, Nawaz S, Hussain A, et al. Ultra-high-molecular-weight-polyethylene (UHMWPE) as a promising polymer material for biomedical applications: A concise review. Polymers (Basel). 2020;12(2):323. doi: 10.3390/polym12020323 32033140 PMC7077409

[18] Afxentiou K, Preidl RHM, Kesting MR, Aksu A, von Bomhard A, Pippich K, et al. Evaluation of ultra-high molecular weight polyethylene (UHMWPE) implants in orbital fracture reconstruction: A multicenter cohort study. J Maxillofac Oral Surg. 2025;24(4):1137–43. doi: 10.1007/s12663-025-02618-w 40756907 PMC12316613

[19] Drakopoulos SX, Psarras GC, Forte G, Martin-Fabiani I, Ronca S. Entanglement dynamics in ultra-high molecular weight polyethylene as revealed by dielectric spectroscopy. Polymer. 2018;150:35–43. doi: 10.1016/j.polymer.2018.07.021

[20] Hussain M, Naqvi RA, Abbas N, Khan SM, Nawaz S, Hussain A, et al. Ultra-high-molecular-weight-polyethylene (UHMWPE) as a promising polymer material for biomedical applications: A concise review. Polymers (Basel). 2020;12(2):323. doi: 10.3390/polym12020323 32033140 PMC7077409

[21] Kozakiewicz M, Szymor P. Comparison of pre-bent titanium mesh versus polyethylene implants in patient specific orbital reconstructions. Head Face Med. 2013;9:32. doi: 10.1186/1746-160X-9-32 24382118 PMC4029398

[22] Brach Del Prever EM, Bistolfi A, Bracco P, Costa L. UHMWPE for arthroplasty: Past or future?. J Orthop Traumatol. 2009;10(1):1–8. 10.1007/s10195-008-0038-y PMC2657354 19384628

[23] Seifert LB, Mainka T, Herrera-Vizcaino C, Verboket R, Sader R. Orbital floor fractures: Epidemiology and outcomes of 1594 reconstructions. Eur J Trauma Emerg Surg. 2022;48(2):1427–36. doi: 10.1007/s00068-021-01716-x 34128084 PMC9001234

[24] Chiang E, Saadat LV, Spitz JA, Bryar PJ, Chambers CB. Etiology of orbital fractures at a level I trauma center in a large metropolitan city. Taiwan J Ophthalmol. 2016;6(1):26–31. doi: 10.1016/j.tjo.2015.12.002 29018706 PMC5602121

[25] Zhang R, Mai Y, Ye H, Lian X, Yang H, Zhu Y, et al. A randomized controlled non-inferiority trial evaluating opioid-free versus opioid-sparing analgesia for orbital fracture reconstruction under general anesthesia. J Pain Res. 2024;17:3707–17. doi: 10.2147/JPR.S491994 39554311 PMC11566577

[26] Asiri M, Aldowah O. Ocular findings in patients with orbital fractures: A 1-year prospective study in a tertiary center. Medicina. 2023;59. 10.3390/medicina59061091 PMC10305705 37374295

[27] Cuevas-Trisan R. Balance problems and fall risks in the elderly. Clin Geriatr Med. 2019;35(2):173–83. doi: 10.1016/j.cger.2019.01.008 30929881

[28] Koryczan P, Zapała J, Gontarz M, Wyszyńska-Pawelec G. Comparison of the results of the treatment of enophthalmos in orbital blowout fracture in children/adolescents and adults. Dent Med Probl. 2021;58(2):179–86. doi: 10.17219/dmp/130815 34036756

[29] Burnstine MA. Clinical recommendations for repair of isolated orbital floor fractures: an evidence-based analysis. Ophthalmology. 2002;109(7):1207–10. doi: 10.1016/s0161-6420(02)01057-6 12093637

[30] Colangeli W, Ferragina F, Kallaverja E, Celano C, Cristofaro MG. Orbital fractures treated in a university hospital of southern Italy: Epidemiology, outcomes and prognostic factors resulting from 538 retrospectively analyzed cases. Oral Maxillofac Surg. 2024;28(3):1219–25. doi: 10.1007/s10006-024-01236-z 38556588 PMC11330396

[31] Committeri U, Arena A, Carraturo E, Austoni M, Germano C, Salzano G, et al. Incidence of orbital side effects in zygomaticomaxillary complex and isolated orbital walls fractures: A Retrospective study in south italy and a brief review of the literature. J Clin Med. 2023;12(3):845. doi: 10.3390/jcm12030845 36769492 PMC9918000

[32] Kavak P, Özdemir M, Kavak N. Injury and clinical patterns according to age groups in pediatric orbital fractures. 2020.

[33] Brucoli M, Arcuri F, Cavenaghi R, Benech A. Analysis of complications after surgical repair of orbital fractures. J Craniofac Surg. 2011;22(4):1387–90. doi: 10.1097/SCS.0b013e31821cc317 21772169

[34] Bartoli D, Fadda MT, Battisti A, Cassoni A, Pagnoni M, Riccardi E, et al. Retrospective analysis of 301 patients with orbital floor fracture. J Craniomaxillofac Surg. 2015;43(2):244–7. doi: 10.1016/j.jcms.2014.11.015 25547215

[35] Homer N, Glass LR, Lee NG, Lefebvre DR, Sutula FC, Freitag SK, et al. Assessment of infraorbital hypesthesia following orbital floor and zygomaticomaxillary complex fractures using a novel sensory grading system. Ophthalmic Plast Reconstr Surg. 2019;35(1):53–5. doi: 10.1097/IOP.0000000000001162 29975327

[36] Rajantie H, Kaukola L, Snäll J, Roine R, Sintonen H, Thorén H. Health-related quality of life in patients surgically treated for orbital blow-out fracture: A prospective study. Oral Maxillofac Surg. 2021;25(3):373–82. doi: 10.1007/s10006-020-00923-x 33280065 PMC8352817

[37] Conforte JJ, Alves CP, Sánchez M del PR, Ponzoni D. Impact of trauma and surgical treatment on the quality of life of patients with facial fractures. Int J Oral Maxillofac Surg. 2016;45(5):575–81. doi: 10.1016/j.ijom.2015.11.022 26723500

